# Early Screen-Time Exposure and Its Association With Risk of Developing Autism Spectrum Disorder: A Systematic Review

**DOI:** 10.7759/cureus.42292

**Published:** 2023-07-22

**Authors:** Saba Sarfraz, Gandhala Shlaghya, Sri Harsha Narayana, Ujala Mushtaq, Basim Shaman Ameen, Chuhao Nie, Daniel Nechi, Iqra J Mazhar, Mohamed Yasir, Ana P Arcia Franchini

**Affiliations:** 1 Research, California Institute of Behavioral Neurosciences & Psychology, Fairfield, USA

**Keywords:** children with asd, asd-like symptom, child's screen time, autism spectrum disorder (asd), screen exposure time, screen time

## Abstract

Autism spectrum disorder (ASD) is a neurological deficit in brain functions that prevents a child from having a normal social life like his peers. It results in the inability to interact and communicate with others. Unsurprisingly, the alarming increase in screen-time exposure in children has become even more of a concern. Electronic devices are a double-edged sword. Despite their benefits, they have many potential hazards to children’s neurological development. Previous studies have investigated the effects of unsupervised screen time and its impact on white matter development during the early years of life of children. The white matter has an important role in the development of neurological functions. This systematic review aims to qualitatively analyze the literature available on early screen time exposure and its association with the risk of developing ASD. This systematic review implemented the Preferred Reporting Items for Systematic Review and Meta-Analyses (PRISMA) 2020 guidelines. PubMed, PubMed Central (PMC), Google Scholar, and Cochrane Library databases were searched for data in the recent six years. A total of 27,200 articles were identified using the MeSH and keywords through four selected databases. Search results revealed 70 from PubMed, 17,700 from Google Scholar, zero from Cochrane Library, and 9,430 from PubMed Central. After applying filters and screening the results by title and abstract and then by full text, 11 studies fulfilled the criteria to be included in the review. We found that the longer the period of screen exposure, the higher the risk that the child may develop ASD. Further, the earlier the child is exposed to screens, the higher the risk of developing ASD in children compared to children exposed later.

## Introduction and background

Autism spectrum disorder (ASD) is a disorder of brain maturation that affects the social skills of children. Children with ASD have difficulties in communication and interacting normally with others [1]. No single cause of autism has been established, but studies suggest a possible combination of genetics and environment [2]. According to Centres of Disease Control and Prevention (CDC), one in 44 children has been diagnosed with ASD, which is four times more common among boys than girls [3]. Autistic people tend to have a lower quality of life than non-autistic people due to social isolation and lack of self-confidence, leading to anxiety and depression [4].

The American Association of Paediatrics recommends one hour per weekday and three hours on weekends for children aged two to five years [5]. It is also advised to avoid unsupervised screen time before two years of age [5]. Unfortunately, the current screen usage surpasses the recommended guidelines [5]. According to an article, most American children exceed the limits of screen-time use, spending anywhere between five to seven hours a day [6]. Moreover, another study in Japan demonstrated that average cell phone use was around 24 hours per week [7].

Unsurprisingly, the alarming levels of increase in screen-time exposure in children have become even more of a concern. The benefits of electronic devices are undeniable, but excessive exposure to screen time has detrimental effects on a child's brain development [8]. A study done in 2019 delved into the effects of extra screen time potentially impacting white matter development in toddlers and preschool children. White matter is responsible for cognitive function, language, and literacy skills [9]. Many studies have found an association between excessive screen usage and autism-like symptoms [10, 11]. Moreover, a study published in 2022 linked prolonged screen-time exposure at one year of age with ASD at three years of age among boys [12]. This could be due to white matter changes, as mentioned earlier, but also neurochemical disruption. Excessive screen light can reduce the production of melanin levels affecting sleep, whereas deficiency of other neurotransmitters, such as dopamine, acetylcholine, gamma-aminobutyric acid (GABA), and 5-hydroxytryptamine (5-HT), was also observed in children who had internet addiction in urban left-behind children, which also causes physical and psychological symptoms [13].

Multiple research projects have demonstrated the negative impacts of screen time, but an interesting study done in 2020 shows better language skills in children with better-quality screen exposure [14]. However, the same study concludes that early exposure to higher screen times has been associated with poor language skills [14]. Another study done in Europe highlights the positive effects of playing video games on young children [15]. Children who played video games had better intellectual functioning and academic performance [15]. We still lack sufficient data to confirm whether any association exists between early screen-time exposure and ASD or ASD-like symptoms, which include poor communication, delay in language development, and odd social interactions.

This systematic review aims to qualitatively analyze the literature available on early screen-time exposure and its association with the risk of developing ASD.

## Review

Methods

Search Source and Search Strategy

This systematic review implemented the Preferred Reporting Items for Systematic Review and Meta-Analyses (PRISMA) 2020 guidelines [16]. PubMed, PubMed Central (PMC), Google Scholar, and Cochrane Library databases were searched for data in the recent six years. Medical Subject Heading (MeSH) was used with keywords screen time, television, and autism spectrum disorder. Using the Boolean "AND" and "OR", the MeSH strategy keywords results being: (("Screen Time"[Majr]) OR "Television"[Majr]) AND ("Autistic Disorder"[Mesh] OR "Autism Spectrum Disorder"[Mesh] ).

Inclusion and Exclusion Criteria

In this systematic review, inclusion criteria included relevance to the question, publishing date within the last six years, full-text articles, papers published only in English language, and papers focusing on preschool and school-going children. Unpublished, grey literature and articles displaying only abstracts were excluded from the study.

Article Screening and Quality Assessment

After filtering the topics on databases, we extracted the relevant articles from the Endnote Citation Manager. Next, we screened the literature and removed duplicates. The remaining articles were screened by title and abstract. We then screened the literature in full text. Finally, we used the Newcastle-Ottawa Scale (NOS) for observational studies and the Joanna Briggs Institute (JBI) checklist for the case report, which satisfied >60%. To assess the quality for risk of bias, three factors were measured: selection, comparability, and outcome.

Four cohort studies were assessed by the NOS. Dehiol et al. [17] and Chen et al. [18] showed good quality, while Melchior et al. [19] and Supanitayanon et al. [20] are of fair quality. The fair quality is because the studies report the time of first exposure to screens where comparability was adjusted to it in addition to hours of exposure to screens. The quality assessment of cohorts is shown in Table 1.

**Table 1:** The quality assessment of the included cohorts according to the NOS. Each * represents a score. * = 1 point and ** = 2 points Note: A study can be awarded a maximum of one star for each numbered item within the Selection and Exposure categories. A maximum of two stars can be given for Comparability.

Author and year	Cohort studies									
	Selection				Comparability	Outcome			Quality score	Quality level
	Representativeness of the exposed cohort	Selection of the non-exposed cohort	Ascertainment of exposure	Demonstration that outcome of interest was not present at the start of the study	Comparability of cohorts based on the design or analysis	Assessment of outcome	Was follow-up long enough for outcomes to occur	Adequacy of follow-up of cohorts		
Dehiol et al. 2022 [17]	*	*	*	*	**	*	*	*	9	good
Chen et al. 2020 [18]	*	*	*	*	**	*	*	*	9	good
Melchior et al. 2022 [19]	*	*	*	*	*	*	*	*	8	fair
Supanitayanon et al. 2020 [20]	*	*	*	*	*	*	*	*	8	fair

Six cross-sectional studies were included in this systematic review. All the studies were of fair quality. No study has described the percentage of non-respondents. The quality assessment of cross-sectional studies is shown in Table 2.

**Table 2:** Quality assessment of the included cross-sectional studies according to the NOS. Each * represents a score. * = 1 point and ** = 2 points Note: A study can be awarded a maximum of one star for each numbered item within the Selection and Exposure categories. A maximum of two stars can be given for Comparability.

Author and year	Cross-sectional studies								
	Selection				Comparability	Outcome		Quality score	
	Representativeness of the sample	Sample size	Ascertainment of exposure	Non-respondents	The subjects in different outcome groups are comparable based on the study design or analysis. Confounding factors are controlled.	Assessment of the outcome	Reporting the results		Level
Alrahili et al. 2021 [21]	*	*	**	*	*	*	*	7	fair
Hu et al. 2019 [22]	*	*	*	*	*	*	*	6	fair
Md Zaki Fadzil et al. 2020 [23]	*	*	**	*	*	*	*	7	fair
Bibi et al. 2022 [24]	*	*	**	*	*	*	*	7	fair
Hill et al. 2020 [25]	*	*	**	*	*	*	*	7	fair
Wu et al. 2016 [26]	*	*	**	*	*	*	*	7	fair

Hu et al. did not use a validated questionnaire, but the questionnaire questions are available and described in the study [22]. The overall appraisal of Dieu-Osika et al. concluded that it is included [27]. The study fulfilled all the criteria of the JBI checklist, except that patients' demographic characteristics are unclear. Therefore, the point of adverse events is not applicable to this case.

Results

Search Outcome

**Figure 1:**
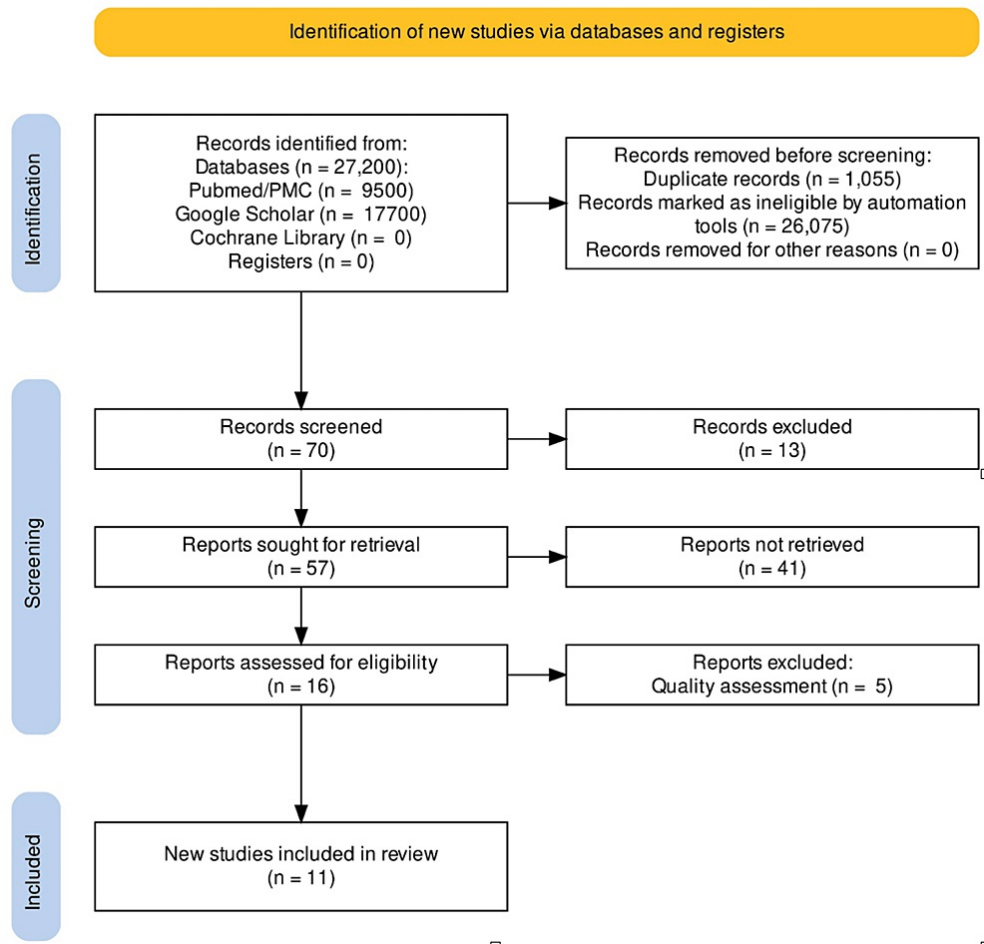
Preferred Reporting Items for Systematic Reviews and Meta-Analyses (PRISMA) flow diagram. The PRISMA flow diagram shows the search process for this systematic review. The databases include PubMed, PubMed Central (PMC), Google Scholar, and Cochrane Library.

A total of 27,200 articles were identified using the MeSH and keywords through four selected databases. Search has revealed 70 from PubMed, 17,700 from Google Scholar, zero from Cochrane Library, and 9,430 from PubMed Central (PMC). After applying filters in all three databases and removing duplicates, we selected 57 articles. The screening was further done by reading the titles and abstracts of these articles. Finally, we selected 16 articles for quality appraisal after applying inclusion and exclusion criteria and full-text availability. A consensus was achieved among the two authors while selecting the articles. The study flow diagram is shown in Figure 1.

Eligible Studies

At the end of the screening process, 11 studies verified the inclusion criteria and were eligible for this systematic review. Six of the 11 included studies are cross-sectional, four are cohorts, and one study is a case report. Eight of the studies were conducted in Asian countries, and three studies were conducted in European countries. The total sample size included in this systematic review is 53,182. This sample includes patients with autism and healthy controls. The age of autism patients ranged from 16 months to six years. The number of males with autism was reported in nine studies. A summary of the general characteristics of the included studies is shown in Table 3.

**Table 3:** Summary of the general characteristics of the included studies. DVD: digital versatile disc; NR: not reported; ADHD: attention deficit hyperactivity disorder; ASD: autism spectrum disorder; SD: standard deviation

Author	Year	Country	Study design	Sample size	Age of the autism group	Males with autism	Screen type	Conclusion
Alrahili et al. [21]	2021	Saudi Arabia	cross-sectional	308	4-6 years	186	mobile phone, tablet/iPad, television	This study demonstrates a significant association between ASD-like symptoms and the amount of screen time/time spent on devices.
Hu et al. [22]	2019	China	cross-sectional	579	mean 5.08, SD = 0.42 years	291	television and DVDs	This study demonstrated that television watching has a detrimental link to the development of math skills, science achievement, executive functioning, and social abilities in Chinese preschool children. Increased television exposure leads to reduced opportunities for active learning and outdoor activities, which are crucial for fostering social interaction, conflict resolution, and handling social challenges among children.
Md Zaki Fadzil et al. [23]	2020	Malaysia	cross-sectional	120	16-30 months	68	NR	Excessive screen media exposure poses a justifiable risk for developing ASD in typically developing toddlers. Children who spend extended hours with screens, especially without adult facilitation, are more likely to be at higher risk of developing ASD. Therefore, parental guidance is essential in determining the appropriate use of electronic screen media for these children.
Dieu-Osika et al. [27]	2019	France	case report	1	25 months	1	Television	Excessive screen time may cause symptoms resembling ASD, but taking a break from media and screens can help alleviate or reverse these symptoms. To achieve positive results, it is crucial to thoroughly remove media exposure and initiate this intervention at an early stage.
Dehiol et al. [17]	2022	Iraq	cohort	107 & 263 control group	5.3 years	88 ASD, 157 control	television (TV) screens, smart mobile phones, tablets, and mixed-use (more than one device)	Exposure to screen devices during early childhood and for extended durations is a crucial factor that triggers the development of ASD. Such devices can have detrimental effects on the developing brains of young children, especially if they are exposed before the age of 2 and for more than 2 hours at a time. Engaging with TV screens and child-oriented content early on significantly increases the risk of developing ASD.
Chen et al. [18]	2020	China	cohort	875 ASD, 28 586 controls	4.33 ± 0.89 years	544	mobile phone, tablet, video games, television, etc.	The study found that starting electronic screen viewing at a younger age, having longer daily screen time, and accumulating more screen time during the preschool period are associated with an increased likelihood of exhibiting autism-like behaviors. Although the study offers preliminary recommendations for supervising electronic screen use in preschoolers, establishing causality would require longitudinal investigations.
Bibi et al. [24]	2022	Pakistan	cross-sectional	100	1-6 years	73	NR	This study managed to establish a relationship between the hours of virtual media exposure and specific behavioral patterns associated with ASD. It is evident from the current research that children exposed to more than 2 hours of virtual media encounter various developmental challenges.
Hill et al. [25]	2020	United States	cross-sectional	120 (ASD 62, ADHD 30, no disorder 28)	36 months	13	television and any other device	In this study, children with concerns for ADHD showed increased screen time and demonstrated a negative correlation between screen time and language development in all the studied groups. Contrary to the hypothesis, children with ASD had intermediate screen time levels compared to the other two groups, and the difference between them was not statistically significant.
Wu et al. [26]	2016	China	cross-sectional	8900	6 months	4710	NR	Pre-schoolers who spend extended periods of time using screens and have insufficient sleep duration are notably more prone to experiencing behavioral issues.
Melchior et al. [19]	2022	France	cohort	12,950	2 years	NR	Tv/computer/tablet/smartphone	This study, conducted on a substantial sample of 2-year-old children, reveals a multifaceted relationship between screen exposure and the risk of ASD.
Supanitayanon et al. [20]	2020	Thailand	cohort	291	6 months - 4 years	NR	television tablets, smartphones, and computers	Pre-schoolers demonstrated improved cognitive development when screen media was introduced later, screen time was managed appropriately, and there was increased verbal interaction during media use in the first 2 years of life.

Discussion

This systematic review included 11 studies that assessed the relationship between ASD and screen exposure. This systematic review's main finding is that the number of hours of screen-time exposure is significantly associated with the development of ASD. Most of the studies included in this review agreed upon this relation and described that these children do not have neurological development of average degree like their peers.

Screen-Time Exposure

Screen-time exposure was reported in nine out of the included 11 studies. Seven studies reported that increased hours spent on different screen types are associated with a higher chance of developmental disorders, such as autistic spectrum disorder. Alrahili et al. [21] reported that high social communication questionnaire scores were significantly associated with screen time (p < 0.05). This reflects that more prolonged exposure to screens is associated with the prevalence of symptoms of ASD. Hu et al. [22] found that most children spent about 2.16 (SD = 1.03) hours of passive screen time, including watching television and videos. In addition, they reported an average of 1.07 (SD = 0.90) hours of active screen time, represented in using computers and smartphones. This reflects that exposure to screens for one to two hours daily could seriously affect the children's neurodevelopment and may result in ASD. Md Zaki Fadzil et al. found that children who spent more than three hours watching screens impend a higher risk of developing ASD according to the M-CHAT-R, a 20-item, parent-report screening tool where they had a mean score of 3 [23]. Children with shorter duration of exposure to screens have a lower risk of developing ASD; here, children who spent less than an hour showed a mean score of 1.56, those who spent one to two hours had a mean score of 1.42, and those who spent more than two hours had a mean score of 0.97. Consequently, screen exposure should be prohibited among children because any duration of exposure is associated with the risk of developing ASD. Dehiol et al. found that patients with ASD spent about four hours daily watching different screens, mainly televisions (p = 0.001) [17]. They also found that the main reason for screen exposure was busy parents who wanted to entertain their children. After these children stopped exposure to screens, there was a significant improvement in their symptoms. We can conclude that parents should find other activities for entertaining their children, rather than letting them watch television or use smartphones. Chen et al. compared children with screen exposure with children who have never been exposed to screens and found that screen exposure is associated with a higher risk of autistic-like behaviors (AOR = 1.61, 95% CI [1.18-2.21]) [18]. Bibi et al. found that ASD children with two hours or more screen exposure have responsive abnormalities, where 48% showed slower responses to ordinary situations and 19% did not respond [24]. Wu et al. reported significantly higher scores on the strengths and difficulties questionnaire in children with higher screen time (p < 0.05) [26].

On the other hand, some studies contradicted our findings. For example, Hill et al. [25] did not find a significant difference between the ASD group and the other control groups, where the control group was not exposed to screens. Moreover, Melchior et al. found that screen time was associated with an intermediate risk of developing neurodevelopmental disorders, but there is no likelihood of being high risk [19].

Alrahili et al. found that 20.4% of the children had issues with social communication, 20.1% had problems understanding non-verbal communication, 34.1% were not interested in their peers, and 32.1% did not have a close friend; in addition, children showed difficulties to gain attention and active communication [21]. Further, Bibi et al. found that children with more than two hours of screen exposure have shown speech delay and difficulties in communication than children with less than two hours of screen exposure [24]. All these findings support and reinforce the findings of this systematic review.

A previous systematic review by Slobodan et al. also investigated the relationship between screen time exposure and the development of ASD [28]. Their findings came in agreement with our findings, where they concluded that children with ASD are more prone to more prolonged screen exposure, which may worsen their symptoms. In this systematic review, the included studies assessed screen exposure before the diagnosis of ASD or before the symptoms appear, while Slobodan et al. assessed screen exposure from the time of diagnosis with ASD [28].

Early Exposure

Three studies reported the relationship between early exposure to different screens and the risk of developing ASD. We found that early exposure is associated with an increased risk of developing ASD than those who are exposed later or never exposed to screens at all during their early years of life.

Md Zaki Fadzil et al. contradicted our findings when they reported no significant relationship between the time of exposure and ASD symptoms (p = 0.432) [23]. On the other hand, Dehiol et al. found a significant relationship between early screen exposure and developing ASD (p = 0.001) [17]. Chen et al. reported that children who were exposed during their first year of life had a significantly higher risk of ASD than other controls (AOR = 2.13, 95% CI [1.54-2.94]) [18].

Kushima et al. found that screen time at one year of age is associated with a higher risk of developing ASD than at three years of age, which may be because, at a younger age, the brain is more prone to genetic variables that affect development later, rather than at three years of age where the brain has already developed some milestones [12]. This further supports our study findings, where early exposure was associated with a greater risk of developing ASD. However, in contrast to our findings, Md Zaki Fadzil et al. found no statistically significant relationship between early screen exposure and the risk of developing ASD [23].

Lin et al., a study that examined the risk of neurodevelopmental conditions concerning screen exposure, found that early exposure leads to sleep disturbances among those children which makes the children more addicted to screens and result in neurodevelopmental disorders [29].

Dieu-Osika et al. described the relationship between early exposure to screens and ASD as "early media overexposure" syndrome, yet they also concluded that ASD symptoms might be reversed by stopping exposure to screens [27].

Limitations and Strengths

The strength of this review is that it includes longitudinal cohort studies; not all the studies are cross-sectional. It also included studies that assessed screen exposure before the development of ASD, which enables us to have a more reliable conclusion about the relationship between screen exposure and the risk of developing ASD.

A critical limitation of this systematic review is cross-sectional studies performed at a single point in time. Longitudinal cohort studies would be more appropriate, especially if they used objective methods for assessing the time of exposure and outcomes. These objective methods may include electronic recording of screen exposure. Additionally, there are so many confounders among the populations of the studies.

For further studies, we recommend including a larger sample size and adjusting all the possible confounders. This will help us in having a more direct causal relationship between screen exposure and the risk of developing ASD.

## Conclusions

To conclude, screens are a critical issue in children's neurodevelopment. They put the children at high risk of developing ASD. The children who are exposed to more screen time than other children showed symptoms of ASD-like difficulties in communication, delayed language skills, delayed cognitive and learning abilities, and inappropriate emotional reactions. Additionally, the exposure of children to screens at an early time in their life makes them at high risk of developing ASD than other children who are exposed later. This is because the first year of life is critical in children's development, and they should be away from exposure to any screen.
